# Supplementary material for: The Endocannabinoid System of the Nervous and Gastrointestinal Systems Changes after a Subnoxious Cisplatin Dose in Male Rats
Source: Pharmaceuticals (Basel). 2024 Sep 24;17(10):1256. doi: 10.3390/ph17101256 (PMC11509924; doi:10.3390/ph17101256)
Supplement: Supplementary file 1 [file pharmaceuticals-17-01256-s001.zip › pharmaceuticals-3184166-supplementary.pdf]

pharmaceuticals

MDPI

Article

The endocannabinoid system of the nervous and gastrointestinal systems changes after a subnoxious cisplatin dose in male rats.

Yolanda López-Tofiño[1,2], Mary A. Hopkins[3,4], Ana Bagues[1,2,5,6], Laura Boullon[3,4], Raquel Abalo[1,2,5,6,7,8*], Álvaro Llorente- Berzal[2,3,4,9*]

1 Department of Basic Health Sciences, University Rey Juan Carlos (URJC), Alcorcón, Spain; yolanda.lopez@urjc.es; ana.bagues@urjc.es; raquel.abalo@urjc.es.
2 High Performance Research Group in Physiopathology and Pharmacology of the Digestive System (NeuGut-URJC), University Rey Juan Carlos (URJC), Alcorcón, Spain; alvaro.llorente@uam.es.
3 Department of Pharmacology and Therapeutics, School of Medicine, University of Galway, Ireland. lboullon@iu.edu; M.HOPKINS9@universityofgalway.ie.
4 Centre for Pain Research and Galway Neuroscience Centre. University of Galway, Ireland.
5 High Performance Research Group in Experimental Pharmacology (PHARMAKOM-URJC), University Rey Juan Carlos (URJC), Alcorcón, Spain.
6 Associated I+D+i Unit to the Institute of Medicinal Chemistry (IQM), Scientific Research Superior Council (CSIC), Madrid, Spain.
7 Working Group of Basic Sciences on Pain and Analgesia of the Spanish Pain Society, Madrid, Spain.
8 Working Group of Basic Sciences on Cannabinoids of the Spanish Pain Society, Madrid, Spain.
9 Department of Physiology, School of Medicine, Autonomous University of Madrid (UAM), Madrid, Spain.
* Correspondence: RA: raquel.abalo@urjc.es; Tel.: +34914888854. ALB: alvaro.llorente@uam.es; Tel.: +34 914 976976

Table S1. Supplementary data. Gene expression of genes related to the endocannabinoid system one week after treatment with saline (n=3-6) or a single dose of cisplatin (i.p., 5 mg/kg; n=2-6) in gastrointestinal tissue (antrum, fundus, ileum and distal colon). The mean percentage of the saline-treated group ($2^{-\Delta CT}$) for saline is expressed as Mean ± SEM of saline- vs cisplatin-treated animals. T-test: * significant difference.

Genes	*cnr1*	*cnr2*	*faah*	*mgll*	*ppara*
Antrum	100.00 ± 45.75 *vs* 102.71 ± 16.39	100.00 ± 57.49 *vs* 79.66 ± 27.09	100.00 ± 32.38 *vs* 161.05 ± 40.86	**100.00 ± 10.84 *vs* 65.70 ± 7.05 ***	100.00 ± 7.02 *vs* 98.32 ± 11.67
Fundus	100.00 ± 71.25 *vs* 47.35 ± 3.70	100.00 ± 26.01 *vs* *81.22* ± 30.19		100.00 ± 18.17 *vs* 101.57 ± 22.28	
Ileum	100.00 ± 87.28 *vs* 57.07 ± 17.16	100.00 ± 22.26 *vs* 65.99 ± 18.64			
Distal colon	100.00 ± 28.26 *vs* 136.66 ± 30.59	100.00 ± 19.48 *vs* 109.06 ± 15.26	100.00 ± 15.68 *vs* 104.40 ± 13.10	100.00 ± 20.61 *vs* 149.57 ± 49.61	

Correspondences: cnr1 corresponds to CB1 receptor; cnr2 corresponds to CB2 receptor; faah corresponds to fatty acid amide hydrolase (FAAH); mgll corresponds to monoacylglycerol lipase (MAGL); ppara corresponds to peroxisome proliferator-activated receptor type α (PPARα)

Table S2. Supplementary data. Gene expression of genes related to the endocannabinoid system one week after treatment with saline (n=4-6) or a single dose of cisplatin (i.p., 5 mg/kg; n=3-6) in nervous tissue (Dorsal root ganglia L4 and L5, Prefrontal cortex, Periaqueductal grey and Amygdala). The mean percentage of the saline-treated group ($2^{-\Delta CT}$) for saline is expressed as Mean ± SEM of saline- vs cisplatin-treated animals. T-test: * significant difference.

Genes	*cnr1*	*cnr2*	*faah*	*mgll*	*ppara*
Dorsal root ganglion L4	**100.00 ± 6.93 *vs* 127.06 ± 8.83 ***	100.00 ± 12.20 *vs* 93.85 ± 10.64	100.00 ± 4.39 *vs* 100.03 ± 7.54	100.00 ± 6.33 *vs* 93.47 ± 8.02	100.00 ± 6.97 *vs* 92.29 ± 9.57
Dorsal root ganglion L5	100.00 ± 8.99 *vs* 127.08 ± 11.72	100.00 ± 15.59 *vs* 78.02 ± 7.65	100.00 ± 6.74 *vs* 102.73 ± 6.16	100.00 ± 5.40 *vs* 99.60 ± 3.99	100.00 ± 3.54 *vs* 95.83 ± 5.50
Prefrontal cortex	100.00 ± 3.05 *vs* 96.19 ± 5.69	**100.00 ± 7.22 *vs* 152.19 ± 8.37 ***	100.00 ± 6.66 *vs* 109.77 ± 8.22	**100.00 ± 3.41 *vs* 113.35 ± 3.61 ***	**100.00 ± 6.98 *vs* 135.28 ± 6.85 ***
Periaqueductal grey	100.00 ± 6.61 *vs* 87.31 ± 8.07	100.00 ± 7.39 *vs* 107.08 ± 8.58	100.00 ± 3.18 *vs* 107.27 ± 5.60	100.00 ± 4.77 *vs* 92.96 ± 7.80	100.00 ± 6.99 *vs* 81.58 ± 7.00
Amygdala	**100.00 ± 2.87 *vs* 87.37 ± 4.27 ***	100.00 ± 13.20 *vs* 86.88 ± 4.05	100.00 ± 4.58 *vs* 102.77 ± 3.54	**100.00 ± 3.51 *vs* 75.34 ± 6.01 ***	100.00 ± 11.14 *vs* 81.72 ± 6.28

Correspondences: cnr1 corresponds to CB_1 receptor; cnr2 corresponds to CB_2 receptor; faah corresponds to fatty acid amide hydrolase (FAAH); mgll corresponds to monoacylglycerol lipase (MAGL); ppara corresponds to peroxisome proliferator-activated receptor type α (PPARα)
